# Data on the Life Cycle Assessment of pizzas cooked and consumed at home taking into account the variability of consumer practices

**DOI:** 10.1016/j.dib.2023.109143

**Published:** 2023-04-12

**Authors:** Adeline Cortesi, Marine Colpaert, Anne Saint-Eve, Bastien Maurice, Gwenola Yannou-Le Bris, Isabelle Souchon, Caroline Pénicaud

**Affiliations:** a Université Paris-Saclay, INRAE, AgroParisTech, UMR SayFood, Palaiseau 91120, France; b Avignon Université, INRAE, UMR SQPOV, Avignon 84000, France

**Keywords:** Life cycle assessment (LCA), Food, Sustainability, Environmental impacts, Industrial ecology, Sustainable food preparation, Sustainable consumer habits

## Abstract

Human food consumption is responsible for significant environmental impacts, which in recent years have been the focus of an increasing amount of research. One of the major results of these efforts has been an appreciation for the ways in which impacts can differ among products. To date, though, relatively little is known about possible differences in the environmental performance of a single food product that is made or produced in different contexts. Furthermore, the influence of consumer practices, such as cooking time or cleaning method, has not yet been investigated. The goals of the study were therefore (i) to compare the environmental impacts of a single food product—in this case, pizza—that is produced in different contexts (industrial, homemade, and assembled at home) and (ii) to investigate the influence of real-world consumer practices on these impacts. Two study models were used: a ham-and-cheese pizza and a mixed-cheese pizza. The functional units (FU) examined were one pizza and 1 kg of ready-to-eat pizza. The system boundaries extended from the agricultural production of ingredients to the consumption of the pizza at home. All inventory data related to the steps occurring before purchase (including storage at the supermarket) came from databases or the literature, while inventory data related to the steps occurring after the sale were obtained from questionnaires answered by 69 consumers who prepared and consumed the six pizza preparation pathways (two recipes multiply three methods of preparation) at home. Background data were selected in the AGRIBALYSE 3.0 and Ecoinvent 3.6 databases. The environmental impacts of the six pizza preparation pathways were calculated by Life Cycle Assessment (LCA) using the characterization method "EF 3.0 Method (adapted) V1.00 / EF 3.0 normalization and weighting set” in SimaPro software. To compare the environmental impacts of the six pizza preparation pathways, 69 LCAs were performed for each; to compensate for missing data from incomplete questionnaires, we performed random draws from the available data to generate the life cycle inventory for each assessment. The data obtained in this study can be used to make recommendations to consumers regarding more environmentally friendly food choices and practices.

**Specifications Table**

**Table:** 

Subject	Environmental Engineering
Specific subject area	Environmental assessment in the food industry
Type of data	Table
How data were acquired	Life cycle inventory (LCI) data were obtained from the scientific literature or generated in this study by (i) direct measurements of product/material weight (balance) and (ii) the answers of 69 consumers to questionnaires related to the preparation and consumption of the six studied pizza preparation pathways. The environmental impacts of the pizzas were calculated by LCA using the “EF 3.0 Method (adapted) V1.00 / EF 3.0 normalization and weighting set” in SimaPro software. Data from the AGRIBALYSE 3.0 and EcoInvent 3.6 databases were used as background data.
Data format	Raw Analyzed
Description of data collection	For the industrial pizzas, the mass of each ingredient was estimated based on the ingredient list and the nutritional values on the packaging. Assembled and homemade pizzas were formulated to be as similar as possible to the industrial versions in terms of nutritional and sensorial characteristics. The mass of the ingredients of the assembled and homemade pizzas was determined using a balance. The masses of all packaging materials were also weighed on a balance. Other life cycle inventory data related to the steps occurring before purchase (ingredient production, ingredient transformation, transport, distribution, and retailing) come from the database AGRIBALYSE 3.0 and from a previous study [1], and were adapted to the weights of our pizzas, ingredients, and packaging. Inventory data related to the steps occurring after purchase come from questionnaires answered by 69 consumers who prepared and consumed the six pizzas at home. The answers used for the data inventory describe the following: Transport between the supermarket and the home: distance between the consumer's home and supermarket and the type of vehicle used.
	Home storage: model and brand of fridge (in order to determine its electricity consumption), how full it was (percentage), and storage time. Dough making: equipment used and use time (for electrical equipment) Tomato sauce making: type of cooking surface (electric or gas) and cooking time. Pizza baking: pre-heating + cooking time. Dish cleaning: cleaning method (handwashing, dishwasher, or both), how full the dishwasher was. Leftovers: mass of leftovers, storage time, reheating time and equipment used (oven or microwave), end of life (consumed or thrown away).
Data source location	Inventory data from France were selected in the Agribalyse 3.0 database for agricultural products. Data from France were selected in the Ecoinvent 3.6 database when possible and complemented by European and global data when necessary. All the inventory and calculated data used in this study are stored in the INRAE research center in Palaiseau (FR).
Data accessibility	Repository name: Data INRAE Direct URL to data: 10.57745/LMBUNB
Related research article	Cortesi, A., Colpaert, M., Saint-Eve, A., Maurice, B., Yannou-Le Bris, G., Souchon, I., Pénicaud, C., 2023. Contribution of consumer practices to the environmental impacts of pizzas. Sustainable Production and Consumption 37, 26–38. 10.1016/j.spc.2023.02.002

**Value of the Data**

• The article presents LCI data related to the home preparation of pizza collected through questionnaires answered by 69 participants.
• The article presents LCIA (Life Cycle Impact Assessment) data for pizzas produced in three different production scenarios: industrial, assembled at home, and homemade.
• These data can be used by LCA experts to describe real-world consumer practices in the home with regard to industrial, assembled, and homemade pizzas.
• These data can also be used to support messaging about good home practices for reducing the environmental impacts of food preparation and consumption.

## 1 Objective

The food cooking and consumption at home, i.e. the steps occurring after product purchase, are poorly documented in the studies dealing with the environmental impacts of food. We generated this dataset to fill this gap. To do so, real world data (mostly distance between consumers home and supermarket, most frequently used transport means to go grocery shopping, home equipment characteristics, use time of each equipment for storage, preparation and cooking of the pizza) were collected through questionnaires answered by 69 consumers who prepared and ate different pizzas. Life Cycle Assessments (LCA) were performed with the collected data. These data were used in a research article [2] to understand: (i) the contribution of food cooking and consumption to the environmental impacts of pizzas, (ii) how the variability of the consumers practices can influence the environmental impacts of pizzas and iii) the differences of the environmental impacts between pizzas produced by different manufacturing methods (industrial, assembled at home, home-made by the consumer).

The present data paper provides the questionnaires used to collect inventory data. These questionnaires could be reused by researchers willing to collect data related to real consumer practices. This data paper also provides the information collected through the questionnaires and the LCA results. This brings transparency to the associated research article. The data are sufficiently described with metadata to be reused by other scientists willing to complete their own data with such information.

## 2 Data Description

The dataset contains files related to the LCIs and LCIAs of two pizzas produced in three different contexts, in which real-world variability in consumer practices is taken into account. It also contains the three questionnaires used during the study.

1. dataset_Questionnaire 1 (french): Questionnaire completed by participants before the study began. It includes questions related to model and brand of different type of household equipment and consumers habits.
1. bis. dataset_Questionnaire 1 (english): English translation of questionnaire 1.
2. dataset_Questionnaire 2 (french): Questionnaire completed by participants after the preparation and consumption of each pizza. It includes questions related to pizza preparation (e.g. pizza baking time), pizza consumption, and leftovers management.
2. bis. dataset_Questionnaire 2 (english): English translation of questionnaire 2.
3. dataset_Questionnaire 3: Questionnaire completed by participants at the end of the study. It includes question related to the consumer preferences and perceptions of the different pizzas prepared.
3. bis. dataset_Questionnaire 3 (english): English translation of questionnaire 3.
4. dataset_pizzas_recipes: Recipes of the six pizzas studied.
5. dataset_pizzas_dough recipes: Recipes of the dough for each pizza.
6. dataset_pizzas_tomato sauce recipes: Recipes of the tomato sauce for each pizza.
7. dataset_pizzas_equipement characteristics: Characteristics of the participants equipment (e.g., power of electrical equipment).
8. dataset_pizzas_equipment use times: Use times of different equipment for pizza making (e.g. oven use time).
9. dataset_pizzas_databases: Type of data used in the study and associated databases.
10. dataset_pizzas_transport: Data related to the types of cars used by study participants and the distances between their homes and the supermarket.
11. dataset_pizzas_packaging: Mass of the different packaging materials for each pizza.
12. dataset_pizzas_cleaning method: Cleaning method used by participants to clean their dishes after each pizza making.
13. dataset_pizzas_number of dish units: Number of dish units cleaned using each cleaning method and for each pizza prepared.
14. dataset_pizzas_leftovers: Data related to the management of leftovers: mass of leftovers, fresh storage time, cooking time, and equipment used for each pizza.
15. dataset_pizzas_LCIA baseline scenario: Baseline LCAs of the six pizzas calculated using the baseline data reported by consumers.
16. dataset_pizzas_LCIA random FU 1 pizza: 69 LCAs performed on each of the six pizzas with a FU of one pizza.
17. dataset_pizzas_LCIA random FU 1 kg: 69 LCAs performed on each of the six pizzas with a FU of 1 kg of pizza.

## 3 Experimental Design, Materials and Methods

For this study, we followed the steps of the LCA methodology [3].

### 3.1 Goal

The goals of this study were (i) to understand the influence of real-world consumer practices on the environmental impacts of a food product, and (ii) to compare the environmental impacts of a product prepared in different contexts (industrial, assembled at home, and homemade). We assessed all of the steps from primary ingredient production to consumption, and identified the main hotspots.

### 3.2 System Definition

Two study models were used: a ham-and-cheese pizza and a mixed-cheese pizza. Both were prepared in three different ways: industrial, assembled at home, and homemade. In total, six pizza preparation pathways were studied: an industrial ham-and-cheese pizza (Ind_H/C), a ham-and-cheese pizza assembled at home (Ass_H/C), a homemade ham-and-cheese pizza (Hom_H/C), an industrial mixed-cheese pizza (Ind_C), a mixed-cheese pizza assembled at home (Ass_C), and a homemade mixed-cheese pizza (Hom_C).

#### 3.2.1 Pizza Selection and Formulation

##### 3.2.1.1 Industrial Pizza Selection

Two industrial pizzas from the French retail market were selected: one ham-and-cheese pizza (Nutri-Score B) and one mixed-cheese pizza (NutriScore D). These two types of pizza were selected because they are the two most popular categories of pizza in France and because we wanted the pizzas to have different nutritional qualities. The assembled and homemade pizzas were then formulated to be similar to the industrial products from a sensory and nutritional point of view (i.e., nutritional composition was identical per 100 g of pizza).

##### 3.2.1.2 Recipe Estimation of Industrial Pizzas

In order to formulate the assembled and homemade pizzas, the first step was to estimate the recipe of the industrial pizzas. To do so, the ingredients and the nutritional composition of the two industrial pizzas were collected from their packaging and entered into an INRAE internal software program called Anatole [4], which provided an estimate of the masses of each ingredient.

##### 3.2.1.3 Formulation of the Assembled Pizza

The pizzas to be assembled at home were made of an industrial dough and an industrial cooked tomato sauce. The first step for the formulation of these pizzas was to identify the industrial dough, tomato sauces, and toppings available in the French retail market. Based on this, the recipe of the assembled pizza was designed to be as close as possible to the estimated recipe of the industrial product in terms of nutritional composition (within the tolerances established by EC regulations n°1924/2006 and 1925/2006). Formulation trials were then carried out in which the recipes of the assembled pizzas were modified in order to produce pizzas that were as close to the industrial versions as possible in terms of nutritional and sensory characteristics (global visual, taste, and texture). The same protocol was used for both pizzas Ass_H/C and Ass_C.

##### 3.2.1.4 Homemade Pizza Formulation

The homemade pizzas were made using a homemade dough and tomato sauce. The first step for the formulation of the homemade pizzas was to identify the ingredients needed to make the dough and the tomato sauce, as well as the toppings available in the French retail market. Based on this, the recipe of the homemade pizza was designed to be as close as possible to the estimated recipe of the industrial product in terms of nutritional composition and sensory characteristics. Formulation trials were then carried out as described above for the assembled pizzas.

##### 3.2.1.5 System Boundaries

The system boudaries included all steps from the agricultural production of ingredients to pizza consumption and waste management. For the industrial pizzas, pizzas were bought at the supermarket and baked at the consumer's home. For the assembled pizzas, the industrial dough and tomato sauce, as well as all the toppings, were bought at the supermarket and assembled and baked at the consumer's home. For the homemade pizzas, the ingredients needed to make the dough and the tomato sauce, as well as all the toppings, were bought at the supermarket. The dough and tomato sauce were prepared by the consumer at home and then used to make the homemade pizza.

### 3.3 Functional Unit

A functional unit of one ready-to-eat pizza was chosen. For the sake of comparison, LCAs were also performed for a FU of 1 kg of ready-to-eat pizza.

### 3.4 Inventory and Data Collection

A panel of 69 consumers was recruited to perform the study. The number 69 was selected as it is the minimum required to perform statistical analysis. The participants were representative of the French population in terms of sex and age. They were recruited through a French agency specialized in sensory analysis located in the Ile-de-France region. The inclusion criteria were as follows: age between 20 and 70 years old, not working in the food industry, not following a particular diet, without any food allergy or intolerance, without any disease that could affect the senses, not pregnant or breastfeeding, in the habit of consuming pizza at least once a month, and able to prepare pizzas at home (in possession of an oven, a refrigerator, and a scale).

The study lasted three weeks in total. Each week, each consumer had to prepare and consume two different pizzas. In order to avoid bias related to the consumption order, the following rules were applied: each week, all consumers prepared and consumed one ham-and-cheese and one mixed-cheese pizza; half of the consumers prepared and consumed the ham-and-cheese pizza first and the other half prepared and consumed the mixed-cheese pizza first; a consumer was not allowed to consume two pizzas from the same production method (industrial, assembled, or homemade) within the same week. The consumers received all dry ingredients the first week of the study, and the fresh ingredients and industrial pizzas were delivered on a weekly basis.

The life cycle inventory of the steps between purchase at the supermarket and consumption was estimated based on participants’ answers to online questionnaires.

#### 3.4.1 Ingredients

The mass of each ingredient in the assembled and homemade pizzas was weighed on a balance (XT 6200C, accuracy = 0.01 g) during the formulation trials. This final mass of each ingredient was given to consumers as part of the recipes so that all 69 consumers could make the same pizzas. Background data from the AGRIBALYSE 3.0 database were used to model the agricultural/food products, adapted to the correct weights. For the emmental and blue cheeses, AGRIBALYSE 3.0 was used to model the impacts of milk, and data from the literature [5] were used to model the cheese-making process.

#### 3.4.2 Packaging

##### 3.4.2.6 Packaging Weights

All packaging materials were weighed using a PRECISA balance (XT 6200C, accuracy = 0.01 g). These data, as well as the data used to model the environmental impacts, are available in the file dataset_pizzas_packaging. The data used to model the packaging waste are described in dataset_pizzas_databases.

##### 3.4.2.7 Packaging Transport

As part of our analysis, we took into account the transportation of packaging materials from the packaging manufacturer to the factory where they were used. We modeled the hypothesis used by the AGRIBALYSE database, in which the transportation of packaging combines road, train, and boat transport as follows:

• Road transport: 350 km for glass, 230 km for other materials.
• Train transport: 39 km for glass, 280 km for other materials.
• Boat transport: 87 km for glass, 360 km for other materials.

#### 3.4.3 Distribution, Retailing, and Transport in Between

The industrial pizzas and the ingredients for the homemade and assembled pizzas were transported to the wholesaler, stored, transported to the supermarket, and stored again until purchase. This transportation, as well as the heat, water, and electricity required to store the products, was taken into account in the LCA using the assumptions for the pizza data of the AGRIBALYSE 3.0 database, adapted to the weight of our products.

#### 3.4.4 Dough Making

The dough-making process was performed at an industrial facility for pizzas Ind_H/C, Ass_H/C, Ind_C, and Ass_C. The electrical consumption associated with this was obtained from Ref. [1] and was adapted to the dough weights of our pizzas. For pizzas Hom_H/C and Hom_C, dough making occurred at home, and the data for our model came from questionnaires answered by the 69 participants of the study. Three dough-making techniques were reported by the consumers: mixing by hand, using an electric mixer, and using a breadmaker. For dough making by hand, no resource flow was calculated. For the two other options, the electrical consumption was included, estimated using Eq. (1).

(1) Energyconsumption(kWh)=Poweroftheequipment(kW)*usetime(h)

The power of the equipment used was reported by the consumers or estimated based on the user manuals of the equipment model reported by the consumers. The equipment use time was also reported by consumers. The related data are available in the file data_pizzas_dough making.

#### 3.4.5 Tomato Sauce Making

Tomato sauce was prepared in industrial facilities for pizzas Ind_H/C, Ass_H/C, Ind_C, and Ass_C. To model this, we used the tomato sauce data from the AGRIBALYSE database: “Cooking, industrial, 1 kg of cooked product/ FR U”. For pizzas Hom_H/C and Hom_C, the tomato sauce was prepared at home, and the data used in the model came from questionnaires answered by the 69 participants of the study. Two cooking options were reported by the consumers: using electricity and using gas. The energy consumption was estimated using Eq. (1). The power of the equipment used was reported by participants or estimated based on the user manuals of the equipment model reported by the participants (dataset_pizzas_equipment characteristics). The equipment use time was also reported in the questionnaire (dataset_pizzas_equipment use times).

#### 3.4.6 Industrial Pizza Making

The inventory data used for the industrial processing of pizzas (for industrial pizzas) came from Ref. [1]. These inventory data were adapted to the appropriate type of pizza and ingredient weights used in this study.

#### 3.4.7 Transportation from the Supermarket to the Home

The following information was requested in the questionnaire:

- Distance from the consumer's home to the supermarket
- Most frequent means of transportation for going to the supermarket
- Frequency of shopping

Most consumers stated that they went grocery shopping once a week. We thus used the hypothesis that this weekly trip to the supermarket allowed the purchase of all of the food items needed to prepare 14 meals (2 meals per day). One pizza represented one meal, or 1/14 of the groceries bought weekly. Thus, an allocation of 1/14 was used to determine the transportation that was attributable to the pizza (or the ingredients needed to make the pizza in the case of pizzas Ass_H/C, Hom_H/C, Ass_C and Hom_C). The model took into account the distance between the consumer's home and the supermarket, as well as the type of vehicle used. The Ecoinvent data chosen for the model are available in dataset_pizzas_databases. The weighting used and the distances are available in the file dataset_pizzas_transport. Due to a lack of available data, hybrids and bioethanol cars were assumed to be gasoline-powered and motorcycles were assumed to be scooters. A round trip was considered.

#### 3.4.8 Home Storage

To model the home refrigeration of pizzas and fresh ingredients, we considered two types of data: data which were the same for all pizzas (electrical consumption of the fridge and reported capacity used) and data which were not the same for all pizzas (volume allocation and storage time). Volume allocations were calculated by estimating the volume occupied by each pizza out of the volume available in the fridge. The annual electrical consumption of each fridge was calculated using technical sheets found online (using the model and brand indicated by the consumers in the questionnaires). Then, the electrical consumption attributable to each pizza was calculated using Eq. (2).

(2) $$\begin{array}{ccc} Electrical\;consumption\;\left(kWh\right) & = & \;\frac{fridge\;annual\;electrical\;consumption\;\left(kWh\right)}{365\;\times \;24}\\ & & \times \;storage\;time\left(h\right)\;\times \frac{Volume\;allocation\left(\%\right)}{filling\;rate\;\left(\%\right)} \end{array}$$

Data on the electrical consumption of consumers’ fridges are available in the file dataset_pizzas_equipment characteristics together with the storage times.

#### 3.4.9 Home Cooking

The brand and model of the electric oven of each consumer, as well as the time used for preheating and baking, were collected *via* the questionnaires. The brand and model of the oven were used to determine its power. The electrical consumption of the cooking step was then estimated using Eq. (1). Oven power are available in dataset_pizzas_equipment characteristics and oven use times are available in the file dataset_pizzas_equipment use time.

#### 3.4.10 Cleaning

The participants were asked to list the dishes they cleaned after preparing, cooking, and consuming the pizzas, as well as the methods they used. Three options for cleaning were reported: hand dishwashing only, dishwasher only, and both. Some consumers reported not cleaning anything. The water consumption, waste water output, and energy consumption were taken into account for the LCA.

For handwashing, the water consumption to clean one dish unit was estimated using online data [6] and multiplied by the number of dish units cleaned. To estimate energy consumption, we used the hypothesis that cold water was 10 °C and that the water heater was able to heat the water up to 55 °C. The energy consumption was then calculated using Eq. (3).

(3) $$Energy\;consumption\;\left(kWh\right)=\;water\;mass\left(kg\right)\;\times Cp\;water\times \;\left(T^{\circ }C\;hot\;water-T^{\circ }C\;cold\;water\right)$$

For handwashing, the energy consumption could be electricity or gas depending on the type of water heater reported by the consumer.

For dishwashers, the participants were asked to report the most common dishwasher program used as well as the model and brand of the dishwasher. The water and electricity consumption were found in the manufacturers’ data sheets.

Weighting was applied based on the number of participants using each cleaning method for each of the six pizzas. The water inputs and outputs, energy consumption, and the number of dish units cleaned, as well as the weightings used, are presented in the files dataset_pizzas_cleaning method and dataset_pizzas_number of dish units. One dish unit represented the dishes used by one person for one meal. The assumptions presented in Table 1 were made.

**Table 1:** Dish unit equivalences.

	Number of dish unit
Plate + knife + fork + glass	1
Large plate	1
Saucepan	2
Large bowl	2
Household robot	2

#### 3.4.11 Leftovers

After consuming the pizza, consumers indicated whether they had any leftover pizza, uneaten crusts, or discarded ingredients. If so, they weighed them and reported what they did with them: threw them away, gave them away, or kept them in the refrigerator to consume later. When leftovers were stored in the refrigerator, consumers were asked to report the following information: storage time, final use (eaten, given or thrown away), and reheating details, if applicable (equipment used (oven or microwave) and heating time). Volume allocations were used to estimate the electrical consumption of the fridge that could be attributable to the leftovers. Electrical consumption was calculated in a similar way as that of pizza storage (Eq. (2)) and pizza cooking (Eq. (1)). The related data are available in dataset_pizzas_leftovers.

### 3.5 Characterization

The LCAs were conducted in SimaPro 9.1.0.11 software using the characterization method "EF 3.0 Method (adapted) V1.00 / EF 3.0 normalization and weighting set”. LCAs were performed for two different FUs (FU = 1 pizza and FU = 1 kg of pizza) in order to investigate how the choice of FU influenced the conclusions. To characterize environmental hotspots, a baseline LCA was performed for each of the six pizzas using average data calculated from consumers’ answers (results available in pizzas_dataset_LCIA baseline scenario). When different practices were used by a subset of the study panel (i.e. using electrical equipment for dough making or mixing the dough by hand), weights were attributed to each practice that represented the proportion of participants using it. Furthermore, for each pizza, 69 LCAs were performed to represent the 69 consumers.

To compensate for missing inventory data from incomplete questionnaires, for each of the 69 LCAs random draws were made of the available data from consumers. LCIA results are available in dataset_pizzas_LCIA_random FU 1 pizza and dataset_pizzas_LCIA random FU 1kg.

## Ethics Statement

This work did not involve human subjects or laboratory animals, and therefore did not encounter any ethical issues.

## CRediT authorship contribution statement

**Adeline Cortesi:** Methodology, Formal analysis, Investigation, Writing – original draft. **Marine Colpaert:** Methodology, Formal analysis, Investigation, Writing – review & editing. **Anne Saint-Eve:** Conceptualization, Methodology, Resources, Funding acquisition, Writing – review & editing. **Bastien Maurice:** Methodology, Formal analysis, Resources, Writing – review & editing. **Gwenola Yannou-Le Bris:** Methodology, Formal analysis, Writing – review & editing. **Isabelle Souchon:** Conceptualization, Methodology, Funding acquisition, Writing – review & editing. **Caroline Pénicaud:** Conceptualization, Methodology, Funding acquisition, Formal analysis, Writing – review & editing, Supervision.

## Declaration of Competing Interest

The authors declare that they have no known competing financial interests or personal relationships which have, or could be perceived to have, influenced the work reported in this article.

## Data Availability

Dataset on the Life Cycle Assessment of pizzas produced in different contexts and with real-world variability in consumer practices (Original data) (Dataverse INRAE).
